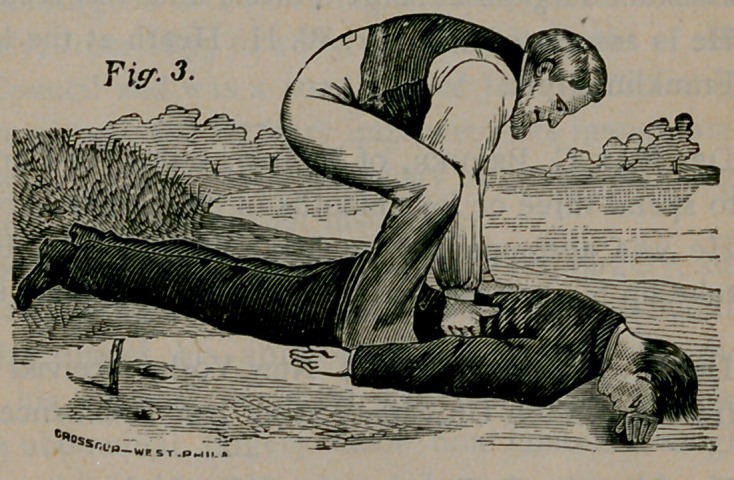# Resuscitation of the Apparently Drowned

**Published:** 1899-07

**Authors:** 


### RESUSCITATION OF THE APPARENTLY DROWNED.

THE season has arrived when accidents by drowning are likely to be common—all too common. It is important, therefore, that all persons likely to witness such accidents should be intelligently informed with regard to the proper measures to be employed for the resuscitation of those who are apparently drowned.

The Michigan State Board of Health has recently published and sent out a circular containing a few well-digested and simple rules on the subject, that are illustrated with wood-cuts, which make the text clear to every person of ordinary intelligence. We think the subject of sufficient importance to reprint the rules and hereby acknowledge our indebtness to Dr. Henry B. Baker, of Lansing, Mich., secretary of the Michigan State Board of Health, for the courteous loan of the cuts. The substance of the circular is as follows :

Rule 1.—Remove all obstructions to breathing. Instantly loosen or cut apart all neck and waist bands; turn the patient on his face, with the head down hill ; stand astride the hips with your face toward his head, and, locking your fingers together under his belly, raise the body as high as you can without lifting the forehead off the ground (Fig. 1), and give the body a smart jerk to remove mucus from the throat and water from the windpipe; hold the body suspended long enough to slowly count one, two, three, four, five, repeating the jerk more gently two or three times. Then act by Rule 2.

Fig 1.

RULE 2.—Keep the patient face downward and maintaining all the while your position astride the body, grasp the points of the shoulders by the clothing, or, if the body is naked, thrust your fingers into the armpits, clasping your thumbs over the points of the shoulders, and raise the chest as high as you can (Fig. 2) without lifting the head quite off the ground, and hold it long enough to slowly count one, two, three. Replace him on the ground with his forehead on his flexed arm, the neck straightened out, and the mouth and nose free. Place your elbows against your knees and your hands upon the sides of his chest (Fig. 3) over the lower ribs and press downward and inward with increasing force long enough to slowly count one, two. Then suddenly let go, grasp the shoulders as before and raise the chest (Fig. 2); then press upon the ribs, etc. (Fig. 3). These alternate movements should be repeated ten to fifteen times a minute for an hour at least, unless breathing is restored sooner. Use the same regularity as in natural breathing.

Fig. 2

Do not give up too soon. You are working for life. Any time within two hours you may be on the very threshold of success without there being any sign of it.

RULE 3.—After breathing has commenced, restore the animal heat. Wrap him in warm blankets, apply bottles of hot water, hot bricks, or anything to restore heat. Warm the head nearly as fast as the body, lest convulsions come on. Rubbing the body with warm cloths or the hand, and slapping the fleshy parts may assist to restore warmth, the circulation of the blood, and the breathing also. The rubbing of the limbs should always be from the extremities toward the body. If the patient can surely swallow, give hot coffee, tea, milk, or a little hot sling. Give spirits sparingly, lest they produce depression. Place the patient in a warm bed, and give him plenty of fresh air; keep him quiet.

Avoid delay. A moment may turn the scale for life or death. Dry ground, shelter, warmth, stimulants, etc., at this moment are nothing — artificial breathing is everything — is the one remedy — all others are secondary.

Fig. 3.

Do not stop to remove wet clothing. Precious time is wasted, and the patient may be fatally chilled by exposure of the naked body, even in summer. Give all your attention and effort to restore breathing by forcing air into, and out of, the lungs. If the breathing has just ceased, a smart slap on the face, or a vigorous twist of the hair will sometimes start it again, and may be tried incidentally, as may also, pressing the finger upon the root of the tongue.

Before natural breathing is fully restored do not let the patient lie on his back unless some person holds the tongue forward. The tongue by falling back may close the windpipe and cause fatal choking.

If several persons are present, one may hold the head steady, keeping the neck nearly straight; others may remove wet clothing, replacing at once clothing which is dry and warm; they may also chafe the limbs, rubbing toward the body, and thus promote the circulation.

Prevent friends from crowding around the patient and excluding fresh air; also from trying to give stimulants before the patient can swallow. The first causes suffocation; the second, fatal choking.

In suffocation by smoke or any poisonous gas, as also by hanging—proceed the same as for drowning, omitting effort to expel water and the like from wind-pipe. In suspended breathing from effects of chloroform, hydrate of chloral, electric shock, and the like, proceed by Rule 2, taking especial pains to keep the head very low, and preventing closure of the windpipe by the tongue falling back.

## Figures and Tables

**Fig 1. f1:**
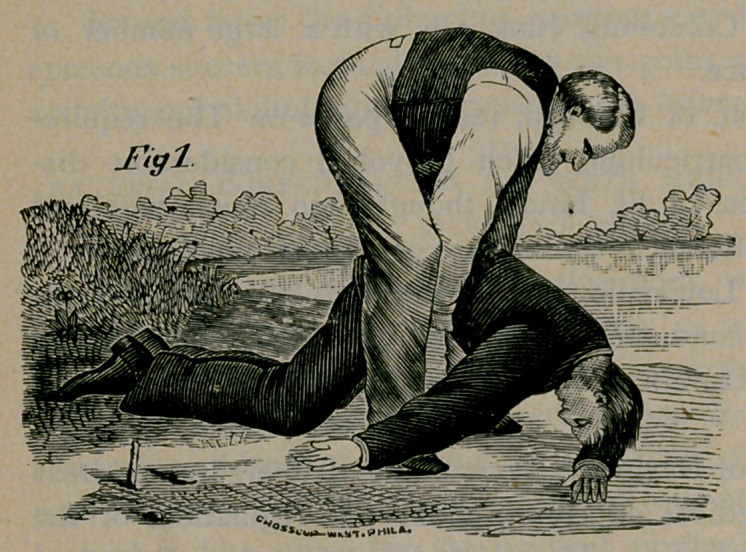


**Fig. 2 f2:**
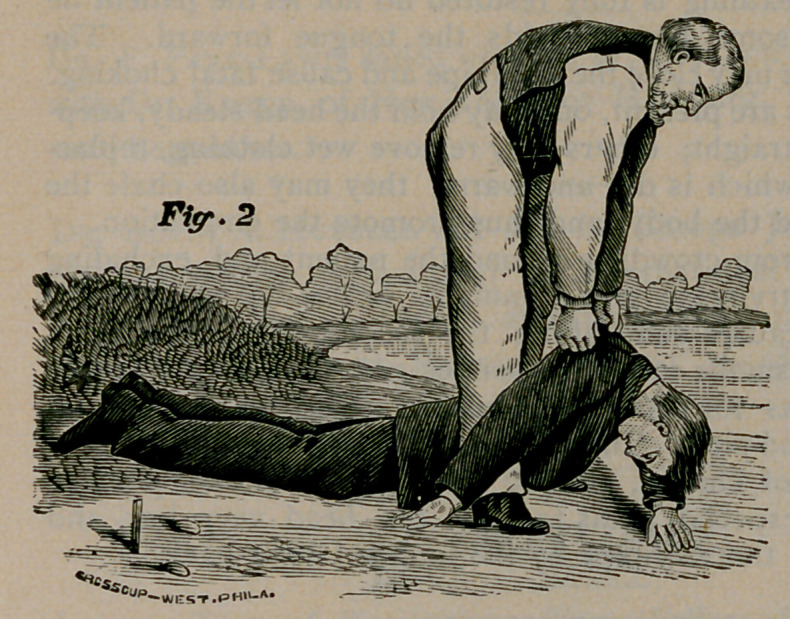


**Fig. 3 f3:**